# Genetic and Ecological Outcomes of *Inga vera* Subsp. *affinis* (Leguminosae) Tree Plantations in a Fragmented Tropical Landscape

**DOI:** 10.1371/journal.pone.0099903

**Published:** 2014-06-16

**Authors:** Oswaldo Cruz Neto, Antonio V. Aguiar, Alex D. Twyford, Linda E. Neaves, R. Toby Pennington, Ariadna V. Lopes

**Affiliations:** 1 Departamento de Botânica, Universidade Federal de Pernambuco, Recife, Pernambuco, Brazil; 2 Department of Wildlife Ecology and Conservation, University of Florida, Gainesville, Florida, United States of America; 3 Royal Botanic Garden Edinburgh, Edinburgh, United Kingdom; USDA- ARS, United States of America

## Abstract

Planting of native trees for habitat restoration is a widespread practice, but the consequences for the retention and transmission of genetic diversity in planted and natural populations are unclear. Using *Inga vera* subsp. *affinis* as a model species, we genotyped five natural and five planted populations in the Atlantic forest of northeastern Brazil at polymorphic microsatellite loci. We studied the breeding system and population structure to test how much genetic diversity is retained in planted relative to natural populations. We then genotyped seedlings from these populations to test whether genetic diversity in planted populations is restored by outcrossing to natural populations of *I. vera*. The breeding system of natural *I. vera* populations was confirmed to be highly outcrossing (*t* = 0.92; *F_IS_* = −0.061, *P* = 0.04), with populations showing weak population substructure (*F_ST_* = 0.028). Genetic diversity in planted populations was 50% less than that of natural populations (planted: *A_R_* = 14.9, *H_O_* = 0.865 and natural: *A_R_* = 30.8, *H_O_* = 0.655). However, seedlings from planted populations showed a 30% higher allelic richness relative to their parents (seedlings *A_R_* = 10.5, parents *A_R_* = 7.6). Understanding the processes and interactions that shape this system are necessary to provide ecologically sensible goals and successfully restore hyper-fragmented habitats. Future restoration plans for *I. vera* must consider the genetic diversity of planted populations and the potential for gene flow between natural populations in the landscape, in order to preserve ecological interactions (i.e. pollination), and promote opportunities for outcrossing.

## Introduction

The long term survival of a species is critically influenced by the maintenance of genetic variation within populations. Habitat loss and fragmentation, and reduced population sizes, decrease genetic diversity within populations, and may increase the levels of inbreeding and population substructure of plant species [1]–[3]. Restoration can mitigate these population changes but the benefits will be limited in plantations of native trees that have been sourced from narrow genetic founding stocks, which have low levels of genetic diversity [4], [5], and as a consequence may suffer from reduced long-term survival and productivity [6]. One way genetic diversity in such plantations may be restored without human intervention is through outcrossing with native conspecific populations [4].

In tropical forests, habitat loss and fragmentation are a ubiquitous consequence of human population growth and the expansion of agricultural areas [7]. One example is the Atlantic forests of Brazil, which once stretched from Rio Grande do Sul to Rio Grande do Norte states, covering an estimated area of 1.3 million km^2^ [8]. The Atlantic rain forest now covers less than 16% of its original extent and occurs in small, isolated fragments [9]. Despite its hyper-fragmented state, the Atlantic rain forest is extremely species-rich with an estimated 13,972 angiosperms species, of which c. 7,000 are endemic [10].

The reported consequences of habitat loss and fragmentation on the reproduction of flowering plants include the reduced range of reproductive attributes of communities, deprived plant-pollinator interactions and decreased gene flow between populations [1], [2], [11], [12], [13], [14]. To mitigate some of the detrimental effects of habitat loss and fragmentation, an increased number of habitat restoration actions have been conducted in several terrestrial ecosystems [2], [4], [15]. Forest restoration in Central and South America, including in Brazil [6], [16], often involves planting single-species stands of *Inga*, with a high density of individuals. Unfortunately, genetic diversity is neither explicitly measured nor considered in some habitat restoration projects [5], [17]. In addition, restored populations are frequently founded with seeds from a limited number of individuals that may only represent a portion of the genetic diversity present in natural populations [4], [18], [19].

Species of *Inga* present brush type-flowers, crepuscular or nocturnal anthesis, and secrete large amounts of nectar [20], [21]. Despite the potentially lower genetic diversity found in restored areas relative to natural areas, planted populations can be considered as a stepping stone for pollinators in animal pollinated plants such as *Inga* spp. [19], and therefore these populations may improve connectivity of natural populations across a fragmented landscape [4]. However, few studies have analyzed the genetic diversity of planted trees in relation to natural populations and the impacts of single species reforestation on gene flow in a fragmented landscape [4], [15], [19].

The aim of this study is to determine the level of genetic diversity in planted relative to natural populations of *I. vera*, and how plantations influence the transmission of genetic diversity from adults to seeds in a fragmented landscape. We test the hypotheses that (*i*) adult individuals in planted populations have reduced genetic diversity relative to adults from natural populations and (*ii*) outcrossing between planted and natural populations increases genetic diversity in seedlings from planted populations relative to their parents. Microsatellite markers revealed remarkable differences in genetic diversity among adults and seedlings of planted relative to natural populations. Additionally, plantations of *Inga* seem to be connected with the natural remnants by pollen flow.

## Materials and Methods

### Study site

**Figure 1:**
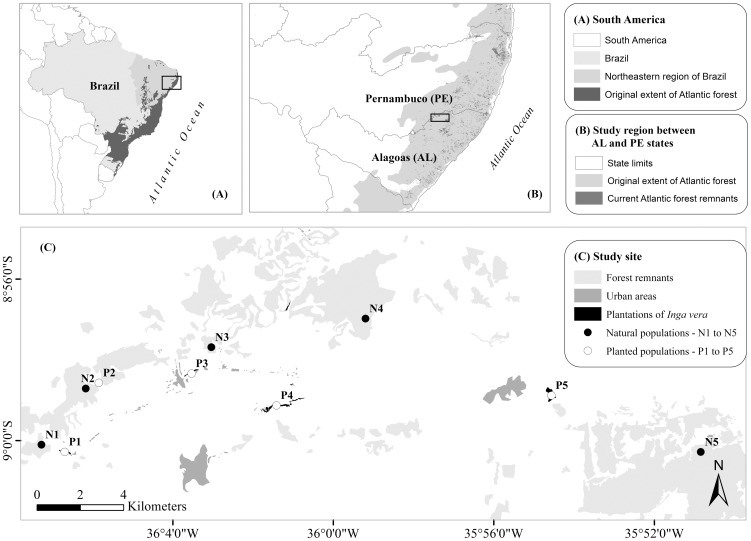
Location of the study area. (A) Map of South America highlighting northeastern region of Brazil and original extent of Atlantic rain forest; (B) Original and remaining distribution of the Atlantic rain forest between Alagoas (AL) and Pernambuco (PE) states; (C) Study site map highlighting remnants of the Atlantic rain forest, *Inga* plantations, and urban areas.

We conducted this study in Usina Serra Grande (USGA), a private property located on the Borborema Plateau, Alagoas state, northeastern Brazil (8°30′S, 35°50′W). Approval to conduct fieldwork was kindly granted by the landowner (L.A. Bezerra) and one of the property managers (C. Bakker). The total area is 24,000 ha, of which ca. 9,000 ha represents Atlantic forest remnants of different shapes and sizes. These remnants are scattered in an agricultural matrix, dominated by sugar cane monoculture and pastures [11], [14] (Fig. 1). In the early 1980's, the landowners reforested areas along river courses as a measure to improve water and soil quality. The reforested patches contain *Inga vera* or *Inga edulis,* trees of which are planted in single species clumps at a density ranging from 20 to 240 (159±89.8) trees per hectare. These monospecific patches are physically isolated from natural areas, and since seed dispersal in *I. vera* is most likely by primates, which are unlikely to cross non-forest areas, there is little chance that any adult *Inga* trees observed in plantations could have arrived via seed dispersal.

The vegetation in the area is classified as moist tropical forest under the Holdridge system, with a dry season from October to February and a rainy season from April to September (climate data for the period 1922–2001, USGA). The richest plant families, in terms of tree species, are Leguminosae (30 spp., among which 14 are subfamily Mimosoideae and eight are *Inga*), Sapotaceae (13 spp.), Lauraceae (11 spp.) and Sapindaceae (8 spp.) [11].

### Study species

*Inga vera* subsp. *affinis* (hereafter *Inga vera*) is distributed from Central America to Uruguay [20]. Its reproductive system is self-incompatible and its fruit set rate is less than 2% in natural conditions [21]. Fruit dispersal is by primates [20]. Though *I. vera* flowers are pollinated by hawkmoths (sphingophilous), they are also visited by bats and hummingbirds [21], (Cruz Neto et al., unpublished). Individuals of *I. vera* flower synchronously and massively at the same period each year [22]. This strategy ensures that the majority of individuals may contribute via pollen and seeds to the seedling cohort. Due to its dependence on biotic pollination, and occurrence in plantations physically isolated from natural remnants, which is a standard restoration practice in northeastern Atlantic forest, *I. vera* is an excellent model species to investigate how planted stands of trees affect gene flow across disturbed landscapes.

### Sampling and data gathering

We randomly selected 10 populations of *I. vera*: five planted populations in the restored monospecific stands, and five natural populations in Atlantic forest fragments (Fig. 1C). We defined a population as individuals from either a forest remnant or planted stand, which were physically isolated in the landscape (Figure 1C). To ensure spatial independence, we selected populations at least 300 m away from each other, both within and among the two spatial treatments (natural and planted). For each population, we randomly sampled 10 reproductive trees totalling 100 adult individuals – 50 for each treatment. We collected six to seven fruit per adult individual, totalling 638 fruits, and sterilized all the 6,416 seeds of these fruits in 1% sodium hypochlorite solution prior to planting in a sterile germination substrate. From the 49 (± 6) available seedlings per maternal family, we sampled five seedlings (i.e. one seedling per fruit), after seedling establishment (ca. 4-month old seedlings). In total, 500 seedlings were sampled across populations. For all the four treatment combinations, two spatial (natural *vs* planted) and two generations (adults *vs* seedlings), we collected young leaves from individuals and stored them in silica gel for DNA extraction.

#### DNA extraction, PCR and genotyping

We extracted DNA from 600 individuals, 100 adults and 500 seedlings, following the Cetyltrimethylammonium Bromide (CTAB) protocol [23]. All individuals were genotyped at four microsatellite loci (Table 1) using primers developed for the related legumes *Pithecelobium elegans* and *I. edulis* [18], [24]. Simplex PCR reactions were performed using a Veriti 384-well Thermal Cycler (Applied Biosystems) in a total volume of 20 µl. The reaction mix contained ∼ 1.5 ng template DNA, 0.32 mM of each primer (the forward was fluorescently labeled with 6-FAM, NED and PET dyes, Table 1), 4 mM KCL-MgCl_2_, 3 mM MgCl_2_, 160 µM of dNTPs, 40 µM of Taq DNA polymerase (Fermentas). We used the following PCR profile: 95°C for 2 min followed by 30 cycles of 95°C for 15 s, 56 or 62°C for 30 s, 72°C for 30 s; 72°C for 15 min (Table 1; Cruz Neto et al., unpublished). PCR products were diluted to 10 ng final concentration with HPLC water. PCR products with each fluorescent dye color were mixed in a single reaction containing 1µl of each diluted PCR product and 6µl of formamide Hi-Di (Applied Biosystems) prior to fragment analysis with a 500-Liz size standard.

**Table 1:** Genetic diversity per locus in adults and seedlings of *Inga vera* in planted and natural populations.

Locus	Reference	T_a_ (°C)	Range (bp)	*H_O_*	*H_S_*	*F_ST_*
*Inga03*	Hollingsworth et al. 2005	56	58–90	0.841	0.807	0.015
						
*Inga08*	Hollingsworth et al. 2005	56	130–174	0.658	0.78	0.052
						
*Inga33*	Hollingsworth et al. 2005	62	216–244	0.583	0.803	0.078
						
*Pel05*	Dayanandan et al. 1997	62	179–215	0.807	0.758	0.072
						

Locus name, reference of the primers, annealing temperature (T_a_), range (bp) of the amplified fragments, observed (*H_O_*) and expected (*H_S_*) heterozygosity, and genetic differentiation coefficient (*F_ST_*). The primers *Inga03* and *Inga33* were labelled with 6-FAM while the primers *Inga08* and *Pel05* were labelled with NED and PET, respectively.

PCR fragments were run on an ABI3730 genetic analyzer (Applied Biosystems). Alleles were scored manually using Peak Scanner 1.0 (Applied Biosystems) and binned according to their motif length with Flexibin [25].

### Data analysis

We tested for Hardy-Weinberg equilibrium (HWE) and linkage disequilibrium for each population using FSTAT v.2.9.3.2 [26], with Bonferroni adjusted significance. We also tested the presence of null alleles using Micro-checker [27] and where necessary applied the INA correction method in Freena software [28].

#### Genetic diversity of adults from planted and natural populations

We compared the genetic diversity of *I. vera* trees between planted and natural populations to test our first hypothesis that genetic diversity is lower in planted than in natural populations. The average number of alleles per locus (*A*), allelic richness rarefied to the minimum sample size of diploid individuals (*A_R_*) [29], and the observed and expected gene diversity (*H_O_* and *H_S_*) [30] values were calculated separately for each planted and natural population using FSTAT. We also scored the number of alleles found exclusively in natural or planted populations using HP-Rare [31].

#### Genetic diversity of adults and seedlings of *Inga vera* stands

The transmission of genetic diversity in planted and natural populations were assessed by comparing estimates of genetic diversity (*A*, *A_R_*, *H_O_* and *H_S_*) between adults and seedlings of each population. Differences between adults and seedlings were tested using a hierarchical analysis with 1,000 permutations implemented in FSTAT, followed by Bonferroni correction.

Seedlings from planted populations were scored for the presence of alleles found exclusively in natural adult populations (see above). The presence of these unique alleles would be consistent with pollen-mediated gene flow from natural to planted populations. By considering unique alleles found in all 50 plants in natural populations against the 50 plants in planted populations, we hope to avoid the upward bias of estimates of private alleles that would be expected given the small sample sizes of individuals per population (10 individuals).

#### Breeding system and population structure

We calculated the inbreeding coefficient (*F_IS_*) [32] for adults and seedlings from each natural population, to gain insight into the breeding system of *I. vera*. We used 10 adults and 10 seedlings (i.e. one seedling per adult tree) in each population for inferring *F_IS_*. Due to the sensitivity of *F_IS_* to small sample sizes, we also calculated the mean across natural and planted stands. Calculations were performed in FSTAT, and significance levels were assessed with 100,000 permutations.

The breeding system of *I. vera* was also inferred through outcrossing rate (*t*) analysis of natural and planted populations. To estimate the outcrossing rate, we used 10 families per population. Families comprised five seedlings from different fruits per tree (i.e. one seedling per fruit). The maternal parent was also genotyped. We used the Bayesian method implemented in BORICE [33] to calculate *t*. We choose a chain of 100,000 steps with a burn-in of the first 10,000 steps to reach a stable posterior distribution of *t* values. The *t* value per population was obtained after five replicate runs.

Two approaches were used to investigate the substructuring of populations. First we calculated the average *F_ST_* [32] across all natural and planted populations. Then, Bayesian clustering analysis in BAPS 5.3 [34] was used to infer the number of genetic clusters for adults from natural populations, and this analysis was repeated for seedlings from natural populations. The software BAPS chooses the most likely number of genetic clusters with a stochastic optimization algorithm. We selected the option ‘clustering of groups of individuals’ and used 10 iterations per clusters (K), with K values ranging from 1 to 5. The output of this initial analysis was used as the basis for the admixture analysis. We selected the option ‘admixture based on mixture clustering’ choosing three as the minimum population size, 100,000 iterations, and 5,000 as the reference number of individuals. Five replicates were made for each K value, and the final number of clusters considered that with the highest log likelihood value. We then inspected the BAPS plots displaying only significant admixture between populations (*P*<0.05).

## Results

### Microsatellite evaluation

All loci were highly polymorphic, containing between 10 and 21 alleles. There was no evidence of null alleles for adult individuals, but there was evidence for null alleles in seedlings of population N4 at locus *Inga 08*. We included this population in subsequent analyses following correction for null alleles, although this had little influence on our results. No evidence for linkage disequilibrium was detected for any planted or natural population, and all populations were in Hardy-Weinberg equilibrium for at least two loci. Measures of genetic diversity (*H_O_*, *H_S_*) were consistent between loci (Table 1).

### Genetic diversity in adults of planted populations relative to natural populations

The total number of alleles across loci in adults of natural and planted populations was 63 and 31, respectively. Twelve alleles were only found in natural populations, whereas no alleles were found exclusively in planted populations.

The average allelic richness per natural population of adults was 7.7 (population range from 7.29 to 8.1; Table 2). In contrast, allelic richness per planted population was low (*A_9_* = 3.7), ranging from 3.42 to 4.26 (Table 2). Comparisons across all planted and natural populations revealed a 50% reduction in allelic richness in adults of planted populations (*A_38_* = 7.599) relative to adults of natural populations (*A_38_* = 14.128, *P* = 0.008; Table 3). Similarly, gene diversity was lower (*P* = 0.013) in planted (*H_S_* = 0.696) than in natural populations (*H_S_* = 0.885; Table 3).

**Table 2:** Genetic diversity per population of adults and seedlings of *Inga vera* in planted and natural stands.

Populations	Adults			Seedlings		
	A	*A_R_*	*H_S_*	A	*A_R_*	*H_S_*
Planted						
P1	4.3	3.62	0.612	6	3.96	0.684
P2	4.8	3.98	0.698	5.75	5.35	0.811
P3	4.0	3.42	0.637	5.25	4.01	0.721
P4	4.3	3.45	0.611	6.25	5.58	0.78
P5	4.8	4.26	0.726	6.5	5.66	0.798
Mean (±SD)	4.4 (0.33)	3.7 (0.36)	0.655 (0.05)	6.01 (0.48)	4.91 (0.85)	0.759 (0.05)
Natural						
N1	8.3	8	0.867	7.5	6.64	0.839
N2	7.8	7.29	0.861	8	7.06	0.881
N3	8.0	7.29	0.838	7.25	6.37	0.831
N4	9.0	8.1	0.885	8.25	7.26	0.886
N5	8.5	7.89	0.876	9.5	7.95	0.897
Mean (±SD)	8.3 (0.48)	7.7 (0.4)	0.865 (0.01)	8.1 (0.87)	7.06 (0.6)	0.867 (0.03)

Average number of alleles per locus (*A*), allelic richness rarefied to nine individuals (*A_R_*; see text) and gene diversity (*H*_S_) per population and overall in adults and seeds among planted and natural populations.

**Table 3:** Genetic variation for the four categories of *Inga vera* population studies.

Populations	*A_R_*	*H_S_*	*F_IS_*	*F_ST_*
Planted				
Adults	7.599^a^	0.696^a^	−0.084^a^	**0.084** ^a^
Seedlings	10.468^b^	0.782^b^	**0.303** ^b^	**0.041** ^b^
Natural				
Adults	14.128^c^	0.885^c^	−0.061^a^	0.028^b^
Seedlings	13.195^c^	0.881^c^	**0.149** ^c^	0.021^b^

Allelic richness (*A_R_*) per locus rarefied to 38 individuals, gene diversity (*H_S_*), inbreeding coefficient (*F_IS_*) and genetic differentiation (*F_ST_*). Values in the same column followed by distinct letters were statistically different at *P*≤0.01 for *A_R_* and *P*≤0.05 for *H_S_*, *F_IS_* and *F_ST_*; All the comparisons were based on 1,000 permutations; Values of *F_IS_* and *F_ST_* in bold were different from zero at *P*<0.05).

### Genetic diversity of seedlings relative to adults

Of the 63 alleles in adults sampled from natural populations, 62 were also present in the seedlings. In contrast, the number of alleles was 38% higher in seedlings (43 alleles) than adults (31) from planted populations. Furthermore, all alleles absent in the adults of planted populations, but present in their seedlings, were found in adults of natural populations. The average allelic richness per population in seedlings from natural stands was 7.06 (population range 6.37 to 7.95), while an average allelic richness of 4.91 (population range 3.96 to 5.66) was found in planted populations (Table 2). The gene diversity in seedlings of planted and natural populations ranged from 0.721 to 0.811 and from 0.831 to 0.897, respectively.

Genetic diversity across planted populations measured as mean allelic richness was higher in seedlings (*A_38_* = 10.468) than in adults (*A_38_* = 7.59, *P* = 0.013; Table 3). Furthermore, gene diversity of seedlings (*H_S_* = 0.782) was higher (*P* = 0.027) than that of adults (*H_S_* = 0.696) in planted populations (Table 3). On the other hand, allelic richness and gene diversity did not differ (*P* = 0.57 and *P* = 0.96, respectively) between adults and seedlings of natural populations (Table 3).

### Breeding system and population structure in *Inga vera*

The population inbreeding coefficient of adults in natural populations was not different from zero (*F_IS_* = −0.061; *P*>0.05), with population values ranging from −0.106 to 0.019. The inbreeding coefficient of seedlings from natural populations, based on one seedling per maternal tree, differed significantly from zero (*F_IS_* = 0.149, *P*<0.05; Table 3) and ranged from −0.067 to 0.243. Despite higher levels of inbreeding in seedlings relative to adults, the average inbreeding coefficient across populations was low (mean *F_IS_* = 0.065, *P*<0.05). In accordance with the low values of *F_IS_*, we detected a high outcrossing rate in natural populations (*t* = 0.92, 2.5 percentile = 0.88, 97.5 percentile = 0.95), ranging from 0.83 to 0.98, as would be expected in species with an outcrossing breeding system. Outcrossing rate in planted populations was also high (*t* = 0.89, 2.5 percentile = 0.81, 97.5 percentile = 0.93).

Adult planted stands exhibited low inbreeding coefficients, which did not differ from zero, similar to that observed in natural populations (*F_IS_* = −0.084, *P* = 0.3). There was no difference between the inbreeding coefficient of adults from planted and natural populations (*P* = 0.659; Table 3). The inbreeding coefficient of seedlings from planted populations was moderate (*F_IS_* = 0.303), and significantly different from zero (*P*<0.05), and higher than *F_IS_* of seedlings from natural populations (*F_IS_* = 0.149, *P* = 0.05; Table 3).

**Figure 2:**
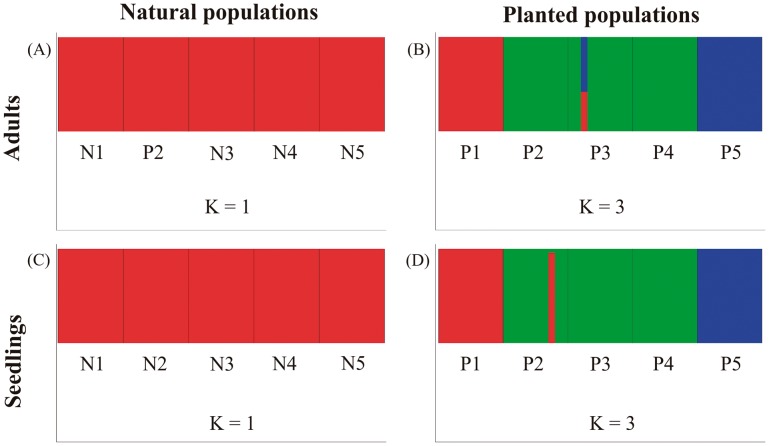
Genetic substructure of planted and natural populations *Inga vera*. Structure plot generated using BAPS 5.3, displaying only significant admixture (*P*<0.05) between planted and natural populations of adults (A and B) and seedlings (C and D). Number of genetic clusters (K) were assessed after five replicates.

The extent of population substructuring was greater in adults of planted populations (*F_ST_* = 0.084) compared to adults of natural populations (*F_ST_* = 0.028, *P* = 0.011; Table 3). Low levels of genetic substructuring were also detected in seedlings from planted populations (*F_ST_* = 0.041; Table 3). Despite the low values, genetic substructuring in adults and seedlings of planted populations was significantly greater than zero (*P*<0.05; Table 3). The results of the Bayesian assignment analyses performed in BAPS were consistent with this pattern, with a single genetic cluster (K = 1) recovered for all adult individuals from natural populations, and three genetic clusters (K = 3) detected in adults from planted populations, with limited admixture between them. The same difference regarding the genetic clustering was detected in seedlings of natural (K = 1) and planted (K = 3) populations, as the respective adults (Fig. 2).

## Discussion

In this study we used *Inga vera* to analyse the retention of genetic diversity in restoration initiatives in the most threatened region of the Atlantic forest of Brazil. We showed that *Inga vera* is an outcrossing species, with high levels of genetic diversity within populations. Comparative analyses revealed that the adults of planted populations have reduced genetic diversity in relation to the adults of natural populations, and also present some genetic structuring, whereas natural populations have no spatial structure. In addition, the seedlings of planted populations have lower genetic diversity than those of natural areas. However, the seedlings of planted populations have higher genetic diversity than the adults, suggesting pollen-mediated gene flow from natural to planted stands. Our results suggest that for self-incompatible species, like *I. vera,* plantations sourced from narrow genetic founding stock may recoup genetic diversity by outcrossing with natural stands. In addition, we hypothesize that plantations of outcrossing species may positively contribute to gene flow through hyper-disturbed landscapes.

### Reduced genetic diversity in planted stands of *Inga vera*

Planted stands of *I.vera* had lower allelic richness and expected heterozygosity relative to natural populations. A reduction of genetic diversity in planted relative to natural populations was also found in *I. edulis* for both nuclear and organellar DNA [5], [18], and this trend has also been observed in other tree species [15], [17]. It is likely that low genetic diversity in *I. vera* planted populations is explained by planting seeds from only a few trees, a standard practice in the northeastern Atlantic forest [16]. However, the increased genetic structure detected in planted populations relative to natural populations, as indicated by higher *F_ST_* values and the greater number of clusters in the Bayesian analysis, suggests that not all planted populations are derived from fruits of a single maternal parent.

The clustering of *Inga* pollen in polyads with approximately 20 grains [35] may also influence patterns of genetic diversity. Each pollen grain in a polyad can fertilize one ovule in the same flower. This strategy, where many pollen grains are dispersed as one unit, and where any one of the grains is able to fertilize the ovules, is called “sweepstake” reproduction [36]. Despite the fact that sweepstake reproduction results in a high seed set for a given fruit, the genetic diversity among seeds of the same fruit will be low [36]. Therefore, the planting of seeds from few fruits will restrict the genetic diversity of planted populations of *Inga* species. We emphasize that restoration efforts with species of *Inga* should be done with seeds from different individuals and fruits to avoid close kinship relationships between the seedlings, which may impair the long-term reproductive output (i.e. seed quality, vigour) of planted populations.

### Recovery of genetic diversity in planted populations through outcrossing

We found evidence suggesting that planted populations might be outcrossing with natural populations. We detected an increase of 30% in allelic richness in seedlings compared with adults of planted populations, while no change was detected in natural populations. The presence of alleles only found in adults from surrounding natural forest fragments (and not in planted stands) provides some support for pollen flow from natural stands enhancing levels of diversity in planted populations. We also detected an increase in expected heterozygosity from adults to seedlings in planted populations, without a change in natural populations, as found for allelic richness. We do, however, recognize that our experimental design, intended to sample multiple populations and seedlings with low coverage of individuals, is unlikely to detect the full range of allelic diversity in a given population, and therefore cannot comprehensively prove outcrossing to natural populations.

Gene flow from natural to planted stands has been detected for *Pinus canariensis*, which is a wind pollinated tree [4]. In this case, immigration rates into artificially regenerated forest were high, ranging from 0.68 to 0.75, and genetic diversity was significantly increased, indicating genetic recovery in *Pinus canariensis* plantations surrounded by larger natural stands. Our results are also consistent with genetic recovery in planted populations of trees, but in this case in an angiosperm tree species that is insect pollinated in a hyper-fragmented area. This shows that while the planting of trees generally results in populations with lower allelic richness [15], [17], [18], as in *I. vera*, genetic diversity can, to some extent, be restored by receiving pollen from natural populations.

### Breeding system and genetic structure of natural populations

Previous pollination experiments with nine *Inga* species, including *I. vera*, demonstrated that all species were obligate outcrossers (have a xenogamic mating system) and are dependent on cross pollination to set fruits and seeds [35]. The uniformly low values for the inbreeding coefficient across natural populations of *I. vera* reported here are consistent with obligatory xenogamy. Furthermore, we found high genetic diversity (e.g. allelic richness) and outcrossing rate values in adults and seedlings from natural populations, as would be expected in an obligate xenogamic tree species with large effective population sizes. Similar patterns of low inbreeding values and high genetic diversity have also been found in *I. edulis* from Peruvian Amazon forest [18]. Overall, the genetic results presented here, and those from previous studies, support obligate xenogamy as the dominant reproductive system for *Inga* species.

The low genetic structuring detected among populations of *I. vera* is likely to indicate high levels of gene flow between populations. Weak population genetic structure may be a consequence of the pollination system of *I. vera* and also confirms the occurrence of outcrossing in the populations that we studied. The majority of *Inga s*pecies, including *I. vera,* can be considered hawkmoth pollinated, despite occasional vistitation by bat and hummingbirds during the day [21], (Cruz Neto et al., unpublished). Hawkmoths, bats and hummingbirds can fly across large areas, ca. 15 km, during their foraging routes carrying pollen grains to distant individuals in well preserved habitats [2], [35], [37]. Fruit and seed set in self-incompatible *Inga* species are ensured by the cross pollination associated with vectors that can move long distances [35]. Therefore, pollen flow between distant individuals in different populations, due to pollinator behavior, contributes to the high outcrossing rate and the weak population substructure found in natural populations of *I. vera*.

Curiously, there is an increase in inbreeding of seedlings relative to adults in both natural and planted populations despite the outcrossing breeding system and low population substructuring in adults of *I. vera*. Habitat fragmentation can alter reproductive strategies in plant communities [11] and negatively interferes in plant-pollinator interactions [12] by favouring short distance dispersion of pollen grains [3]. These changes can increase inbreeding by leading to crossing between related individuals [3], [13]. Our study site is included in the Pernambuco endemism centre which is the most fragmented region of Atlantic rain forest [40].

Despite their large foraging area, hawkmoth species are negatively affected by habitat disturbance and may become locally extinct, or have reduced foraging routes in hyper-fragmented habitats [38], [39]. This could also reduce pollen flow between populations, and increase breeding among potentially related individuals within the same forest fragment. Thus the association between intense forest fragmentation in this area and the dependence of biotic pollinators may contribute to the increase in inbreeding of seedlings of *I. vera* in both natural and planted populations.

### Implications for the conservation and restoration of the Atlantic forest

Many habitat restoration plans have been implemented in the Atlantic forest during the last 30 years [16]. The current consensus is that restoration plans must enhance natural succession and preserve ecological interactions, such as pollination services that may be critical for ensuring restoration success [41], and high genetic diversity of planted populations [17]. Plants in tropical biodiversity hotspots are likely to exhibit high levels of biotic dependence for pollination [41], [42]. We emphasize that the success of restoration actions in hyper-fragmented ecosystems, such as the Atlantic forest, is likely to involve high number of plant species pollinated by vectors with the ability to fly long distances in their foraging routes [41]. This is the case of species of *Inga* in which the main pollinators are typically hawkmoths, bats and hummingbirds [20], [22], [35], [43]. Pollination in reforested areas may be also facilitated by proximity to natural patches that support pollinator communities [41], such as some of the reforested stands in this study.

The genetic recovery found in this study may also be attributed to the highly synchronous flowering among populations of *I. vera* in the northeastern Atlantic forest [22]. The synchronous flowering events of *I. vera* which are typical of other *Inga* [22], [44] species, means that planted populations may be an abundant source of flowers for pollinators supplementing natural populations. Planted stands of *I. vera* with a high density of individuals can increase the availability of floral resource such as nectar and pollen, and therefore favour the attraction of pollinators [45], [46], connecting natural fragments with the reforested stands.

## Conclusions

Our population genetic data for *Inga vera* reveal that low genetic diversity of planted populations can be recovered by cross-pollination with natural stands, particularly given the outcrossing nature of the study species. Recovery of genetic diversity will, however, be hampered if the genetic diversity of natural stands is reduced by habitat degradation, or if very large stands are planted using a narrow genetic founding stock. Future restoration plans in the Atlantic rain forest should consider the potential of pollen dispersal across large spatial scales, and make an effort to ensure high genetic diversity in planted populations, in order to facilitate successful restoration in this hyper-fragmented ecosystem.
